# Innovative Dentin Remineralization and Tubule Occlusion Using Titanium Dioxide–Doped Phosphate Glass and Diode Laser: An In Vitro Study

**DOI:** 10.1055/s-0045-1814463

**Published:** 2026-01-19

**Authors:** Dawood Alshetiwi, Ahmad Alkhalaf, Abdulwahab Alkhamees, Mohammad A. Mohammad, Naser Alotaibi, Saaid Al Shehadat, Ensanya A. Abou Neel

**Affiliations:** 1 Department of Oral and Craniofacial Health Sciences, College of Dental Medicine, University of Sharjah, Sharjah, United Arab Emirates; 2 Department of Restorative Dentistry, College of Dental Medicine, University of Sharjah, Sharjah, United Arab Emirates; 3 Biomaterials and Tissue Engineering, UCL Eastman Dental Institute, Royal Free Hospital, London, United Kingdom

**Keywords:** dentin hypersensitivity, remineralization, acid challenge, phosphate glass, diode laser

## Abstract

**Objective:**

Titanium dioxide-doped phosphate glass (TDPG) is silica-free with enhanced chemical stability and ion release properties that could help in dentin remineralization. There is no evidence, however, regarding whether diode laser irradiation can enhance the clinical performance of TDPG paste in this regard. This article aims to evaluate the in vitro effectiveness of the combined use of TDPG paste and diode laser in occluding dentinal tubules, promoting dentin remineralization, and resisting acid challenge in comparison to a commercially available fluoride-containing NuPro prophy paste.

**Objectives:**

This article aims to evaluate the in vitro effectiveness of the combined use of TDPG paste and diode laser in occluding dentinal tubules, promoting dentin remineralization, and resisting acid challenge in comparison to a commercially available fluoride-containing NuPro prophy paste.

**Materials and Methods:**

Seventy mid-coronal dentin discs were prepared from extracted human premolar and molar teeth and randomly distributed into seven groups ( *n* = 10): sound dentin (control), etched dentin (negative control), etched dentin + diode laser, TDPG, TDPG + diode laser, NuPro, and NuPro + diode laser. Treatments were applied accordingly, followed by a 6% citric acid challenge to assess acid resistance. Scanning electron microscopy (SEM) and Raman spectroscopy (RS) were used to evaluate dentinal tubule occlusion and quantify mineral content [phosphate (960 cm ^-1^ ) and carbonate (1,070 cm ^-1^ )] before and after the acid challenge, respectively.

**Results:**

SEM analysis showed incomplete tubules' occlusion with TDPG and a more uniform surface coverage with NuPro. Laser irradiation improved surface sealing for both materials. After the acidic challenge, NuPro + laser demonstrated better surface integrity, while TDPG + laser showed moderate resistance. RS revealed a significant reduction in the intensity of the phosphate peak after acid exposure in the NuPro groups, particularly with laser (45.8 and 48.9%, respectively), despite the initial high intensity in comparison to a reduction of 1.8% in the TDPG + laser group. TDPG-treated groups demonstrated increased phosphate and carbonate intensities post-acid challenge. Carbonate intensity followed similar trends, confirming greater acid resistance of TDPG formulations compared with NuPro.

**Conclusion:**

TDPG paste demonstrated a more chemically stable remineralization process, while NuPro prophy paste achieved better surface integrity after acidic challenge when used with a diode laser, but laser-induced damage in the form of microcracks was evident. The use of diode lasers may enhance the interaction and penetration of desensitizing agents, although compatibility with material composition must be improved for long-term performance.

**Clinical Significance:**

For immediate symptomatic relief of dentin hypersensitivity, a dual strategy using NuPro + laser is indicated. However, to achieve a sustained mineral reinforcement, the combined use of TDPG-based therapies with laser is recommended.

## Introduction

Dentin hypersensitivity (DH), a common oral condition, shows wide variation between 1.3 and 92.1% and primarily affects people within the 20- to 40-year age range. 1 Recent global meta-analyses estimate an average prevalence of 11.5% in the general population, with substantially higher rates (60–98%) reported among patients with periodontal disease. The condition significantly impacts patients' quality of life, affecting daily activities such as eating, drinking, and oral hygiene practices, leading to considerable psychosocial and economic burden. 1 2 3 4

Pursuant to the hydrodynamic theory and prior research, decreasing dentin permeability through occlusion of dentinal tubules is an efficient treatment for dentin hypersensitivity. 5 6 However, commercial desensitizing products demonstrate suboptimal stability and high recurrence rates (6–75%), with many requiring weeks to achieve efficacy. 5

Current agents include casein phosphopeptide-amorphous calcium phosphate (CPP-ACP), 7 bioactive glass (e.g., NovaMin) 8 and fluoride-based compounds 9 (e.g., Sensodyne Repair and Protect), necessitating the development of novel materials with improved long-term stability. Phosphate glasses represent an innovative bioactive class extensively studied for hard tissue regeneration. 10 Titanium-dioxide doped phosphate glass (TDPG), composed of phosphorus pentoxide, calcium oxide, and sodium oxide, exhibits enhanced chemical stability through TiO _2_ incorporation (3–7 mol%), tunable ion release properties, and demonstrated remineralization potential without silica-related biocompatibility concerns. Recent evidence by Abou Neel et al confirmed favorable remineralization outcomes using a TDPG-polyacrylic acid paste on demineralized dentin. 11

Laser-assisted DH management has attracted significant attention since its introduction in 1985. 12 Diode lasers (810–980 nm) have become the preferred choice due to superior cost-effectiveness, portability, and ease of operation compared with Nd:YAG or Er:YAG systems. 12 13 These lasers achieve tubule sealing through protein coagulation and partial peritubular dentin melting via non-contact application at 0.7 W in continuous mode. 14

Combining diode lasers with bioactive materials may synergistically enhance dentin surface energy, accelerate ion exchange kinetics, and improve mineral nucleation potential through thermal-chemical interactions. 11 Morphological assessment via scanning electron microscopy (SEM) and molecular analysis via Raman spectroscopy complement each other. SEM qualitatively evaluates dentinal tubule occlusion and crystalline deposits, while Raman spectroscopy quantitatively assesses remineralization through phosphate (PO _4_^3−^ ) and carbonate (CO _3_^2−^ ) peak intensities. 15

To our knowledge, no previous studies have evaluated titanium dioxide-doped phosphate glass combined with diode laser treatment for dentinal tubule occlusion and remineralization. This article aimed to (1) compare dentinal tubule occlusion and remineralization effects of TDPG and NuPro prophy paste with or without diode laser treatment and (2) assess changes following 1-minute exposure to 6% citric acid challenge, simulating dietary acid exposure and evaluating material durability. 16 17 18 The null hypotheses were: **H _01_** : There is no difference in dentinal tubule occlusion (measured qualitatively by SEM) and remineralization (measured quantitatively by phosphate and carbonate peak intensities) between TDPG paste and NuPro prophy paste with or without laser. **H _02_** : There is no difference in dentinal tubule occlusion and remineralization following acidic challenge among treatment groups.

## Materials and Methods

### Ethical Approval and Sample Size

This study was approved by the University of Sharjah Research Ethics Committee (REC-24-01-26-11-S) and conducted in accordance with the declaration of Helsinki. A power analysis was conducted using G*Power software (version 3.1.9.7, Heinrich-Heine-Universität Düsseldorf, Germany) and with parameters: effect size *d* = 0.8, *α* = 0.05, power = 0.90). The analysis indicated a minimum sample size of 63 specimens based on quantitative Raman spectroscopy outcomes reported in data from previous studies. 19 20 Considering potential sample loss during preparation and analysis, the total sample size was set at 70 dentin discs ( *n* = 10 per group).

### Sample Selection and Preparation

Thirty-five leftover human extracted premolar and molar teeth belonging to healthy patients (aged 18–55 years) were collected from the Oral and Maxillofacial Surgery Clinic of the College of Dentistry, University of Sharjah. Teeth were extracted for orthodontic, periodontal, or prosthetic reasons unrelated to this study. The inclusion criteria were: sound, non-carious teeth without evidence of cracks or developmental malformations. Meanwhile, the exclusion criteria involved coronally restored teeth with or without previous root canal treatment, as well as teeth presenting with hypoplasia, hypomineralization, and fluorosis. Premolars and molars were selected because they provide adequate midcoronal dentin thickness (typically 2–3 mm) necessary for sectioning 1.5 mm discs to provide representative dentin samples. Teeth were disinfected in 10% buffered formalin solution (pH: 7.0) for 48 hours. Then, teeth were rinsed thoroughly with distilled water and stored in fresh 10% buffered formalin solution at room temperature (20–25°C) for a maximum of 30 days before specimen preparation.

Seventy mid-coronal dentin discs ( *n* = 70) of 1.5 mm thickness were prepared by cutting teeth mesiodistally along the cementoenamel junction using a water-cooled low-speed diamond saw (Isomet 1000 Linear Precision Saw; Buehler Ltd, Illinois, United States) at a blade speed of 400 rpm to avoid heat generation. Discs were measured with a digital micrometer (Mitutoyo Corporation, Japan) after each cut, and all procedures were performed by a single operator.

### Randomization and Group Allocation

Dentin discs were numerically labeled in ascending order from 1 to 70. Randomization was performed using Microsoft Excel software (version 2304, build 16.0, Microsoft Corporation, Redmond, Washington, United States) utilizing the RAND function. Random numerical tags were generated against the entered specimen labels, followed by sorting from smallest to largest values to obtain the randomized allocation sequence. The samples were distributed into seven experimental groups, with each group containing 10 samples ( *n* = 10) as summarized in Table 1 .

**Table 1:** Summary of different groups used in this study

Group	*n*	Description
Group 1	10	Sound dentin
Group 2	10	Etched-dentin
Group 3	10	Etched-dentin with diode laser
Group 4	10	TDPG paste on etched dentin
Group 5	10	TDPG paste on etched dentin with diode laser
Group 6	10	NuPro prophy paste on etched dentin
Group 7	10	NuPro prophy paste on etched dentin with diode laser

### Blinding Procedures

A single operator was assigned to apply the treatments and was blinded to subsequent analysis outcomes but aware of treatment allocation (single-blinding). SEM and Raman spectroscopy analyses were performed by independent examiners blinded to group allocation.

### Surface Etching Protocol

One surface of each sample was marked using a permanent marker to specify the working surface. Groups 2 to 7 were etched with 37% phosphoric acid (Scotchbond Universal Etchant; Solventum, St. Paul, Minnesota, United States) gel for 15 seconds using a micro brush, rinsed thoroughly, and gently dried. For Groups 4 and 5, TDPG paste was prepared by mixing powder of the material with 10% polyacrylic acid (GC Dentin Conditioner, GC, Tokyo, Japan) at a powder-to-liquid ratio of 2:1 by weight on a glass slab to produce a thin paste consistency. In Groups 6 and 7, NuPro prophy paste (DENTSPLY Professional, York, Pennsylvania, United States) was applied directly from the manufacturer's container as a thin, uniform layer using a clean microbrush, burnished for 3 seconds, then left to dry for 60 seconds at ambient temperature.

### Diode Laser Application

Laser treatment was performed using an 810 nm diode laser system (Medency Primo, Vicenza, Italy), 300 μm optical fiber tip with a yellow-coded handpiece to concentrate laser energy over a clinically relevant small area. The Medency Primo diode laser complies with IEC 60825–1 laser safety standards. The wavelength 810 nm was selected instead of 980 nm because it exhibits better absorption by pigmented dentin components with lower scattering and less pulpal temperature rise, offering efficient tubule sealing at moderate output power. 21 22 Laser output was verified using a calibrated power meter (Thorlabs PM100D, Thorlabs Inc., Newton, New Jersey, United States). The fiber tip was positioned in non-contact mode, perpendicular to the dentin surface. Irradiation protocol utilized the pre-set “Desensitize” mode with an output power of 0.8 W in continuous wave mode (CW), delivering three cycles of 30 seconds active irradiation followed by 10-second pauses, resulting in 24 J of energy per cycle (0.8 W × 30 second and total energy of 72 J per specimen. The selected power and mode balance therapeutic efficacy for protein coagulation and peritubular dentin melting without causing irreversible pulpal damage or excessive surface charring. The operator wore wavelength-specific laser safety eyewear (optical density OD 5+ for 800–1,000 nm; NoIR Laser Company, model CYN-08, Plymouth, Michigan, United States) conforming to EN 207 standards. 23 24 Parameters align with published literature demonstrating effective dentinal tubule occlusion. 21 22

### Citric Acid Challenge

Following treatment application, three specimens per group ( *n* = 3) were randomly selected using a random number generator and submitted for acid challenge, which was conducted following a protocol mentioned in previous studies. Specimens were statically immersed in 6 % citric acid (w/v) for 1 min, followed by rinsing with distilled water and gently air-drying. The 1-minute exposure models brief dietary acid episodes typical of human consumption, providing realistic yet controlled erosion simulation in accordance with ISO/TR 14569–2 recommendations for erosive challenges. 25 Citric acid was preferred over lactic or hydrochloric acid because it is a predominant dietary acid and acts as a calcium-chelating agent, providing a reproducible and clinically relevant dissolution stress. A single exposure was used to model a typical erosive episode rather than cumulative wear.

### Scanning Electron Microscopy

Two representative specimens from each group ( *n* = 2), before and after acid challenge, were selected for SEM evaluation. Dried specimens were mounted on aluminum SEM stubs, sputter-coated with gold (Au) alloy using a Quorum Technology Q150T-S Sputter Coater (Quorum Technologies Ltd., Lewes, East Sussex, United Kingdom). Gold was used instead of carbon to enhance secondary electron emission, providing superior surface resolution. Imaging was captured at 10 kV accelerating voltage, working distance  ≈ 10 mm, magnifications  × 2,000 and  × 5,000 ; three fields per specimen were obtained. Assessment criteria included: (1) dentinal tubules' occlusion status: open versus closed, based on complete absence or presence of paste precipitates over the tubules' orifices (2) uniformity of surface coverage; (3) presence of crystalline deposits; (4) surface morphology characteristics (smooth, rough, melted); and (5) presence of defects (cracks, voids, debris). Given the limited number of specimens examined by SEM ( *n* = 2 per group), these images were used solely for qualitative morphological assessment rather than quantitative comparison. The observations are intended to illustrate representative surface features characteristic of each treatment condition.

### Raman Spectroscopy

Three representative specimens from each group ( *n* = 3) were submitted for Raman spectroscopy before and after acidic challenge using a Renishaw InVia system with a 785-nm excitation laser. Two spectra were obtained from each specimen at different regions of the dentin disc. The 785-nm wavelength was selected to minimize fluorescence from organic dentin components and prevent thermal alteration, providing optimal signal to noise. Spectral resolution was 2 cm ^−1^ ; baseline correction was performed. Data analysis employed WiRE v5.3 and OriginPro 2021. Peak assignments at 960 cm ^−1^ (PO _4_^3−^ ) and 1,070 cm ^−1^ (CO _3_^2−^ ) were confirmed with established dentin standards from published Raman studies, ensuring comparability. 26 27

### Statistical Analysis

All statistical analyses were performed using SPSS Statistics software (version 28.0, IBM Corporation, Armonk, New York, United States) with a significance level set at *α* = 0.05. Data were first assessed for normality using the Shapiro–Wilk test and homogeneity of variance using Levene's test, and data were found to be normally distributed. For Raman spectroscopy peak intensity data, one-way ANOVA was used to compare mean differences among the experimental groups; with Tukey's honest significant difference (HSD), post hoc test was applied for pairwise multiple comparisons. Comparisons of phosphate and carbonate intensities before and after the acid challenge were performed on independent sample subsets rather than repeated measures, as different specimens were used for pre- and post-challenge analyses. A schematic overview of the experimental workflow is presented in Fig. 1 .

**Fig. 1:**
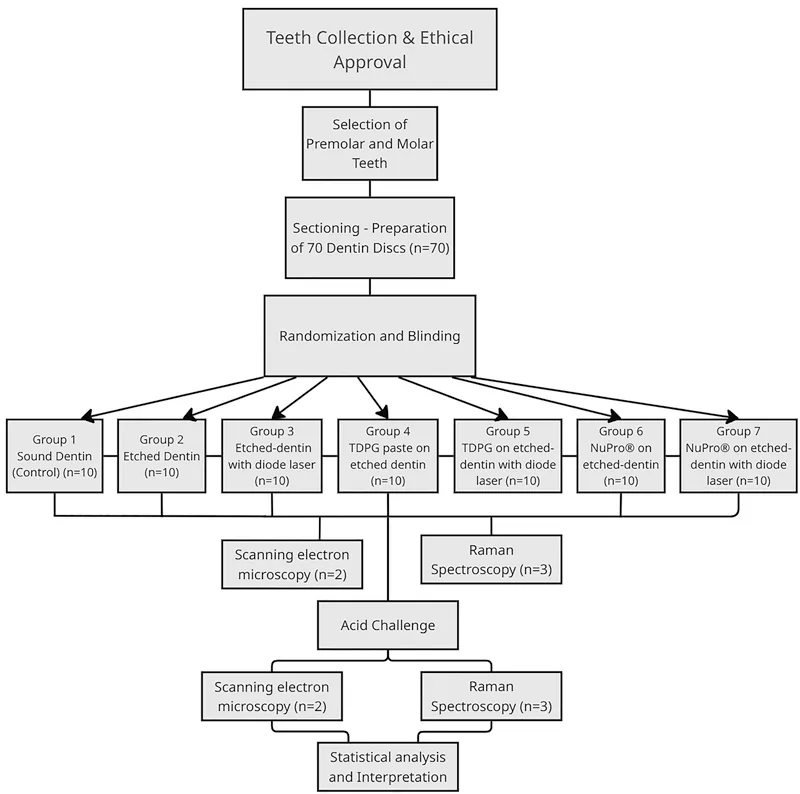
A schematic overview of the experimental workflow.

## Results

### Scanning Electron Microscopy Observations

Fig. 2 presents representative SEM micrographs of etched dentin and dentin treated with TDPG and NuPro prophy pastes, illustrating characteristic surface patterns observed in the examined specimens. After etching, the smear layer and plugs were removed, and the dentinal tubules were completely opened ( Fig. 2A , B). Samples treated with TDPG ( Fig. 2C, D ) or NuPro prophy paste ( Fig. 2G, H ) showed the presence of irregularly shaped precipitates that cover the dentin surface. After the acidic challenge, the precipitates were removed, leaving completely opened tubules, particularly for the TDPG group ( Fig. 2E, F ), but some tubules remained completely closed with the NuPro treated group ( Fig. 2I, J ).

**Fig. 2:**
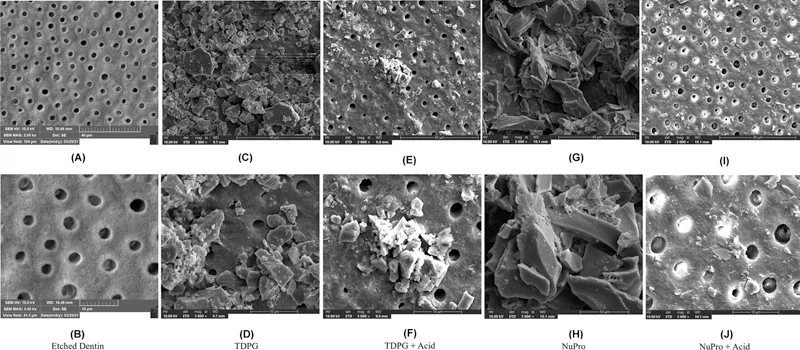
Representative SEM microimages under ×2,000– × 5,000 magnification showing samples of etched dentin ( **A, B** ), treated with TDPG before and after acidic challenge with 6% citric acid ( **C–F** ), NuPro before and after acidic challenge ( **G–J** ). After the acid challenge, both materials exhibited partial loss of surface deposits, with TDPG showing moderate resistance and NuPro displaying greater surface disruption. Scale bar = 40 µm for ×2,000, 10 μm for ×5,000.

Fig. 3 represents the SEM micrographs for different groups treated with laser before and after acidic challenge. The application of laser alone produced obliteration of some dentinal tubules' orifices, but with evidence of micro-cracking and melting of dentin seen on the superficial surface ( Fig. 3A, B ). After the acidic challenge, many dentinal tubules were completely opened ( Fig. 3C, D ). In both groups, where TDPG and NuPro prophy pastes were applied and followed by diode laser application, the underlying pattern of dentin was completely concealed and replaced by a melted and wavy appearance resulting from the laser irradiation and effective penetration of the occluding pastes, without evidence of microcracks ( Fig. 3E–H ). After the acidic challenge of TDPG paste with laser-treated samples, many tubules appear widely opened, suggesting significant erosion or dissolution of the TDPG layer, with the presence of small deposits within dentinal tubules and on the intertubular dentin ( Fig. 3I, J ). For NuPro prophy paste and diode laser-treated samples, less exposure of dentinal tubules and a more intact and uniform surface layer was observed, but with more micro-cracks ( Fig. 3K, L ).

**Fig. 3:**
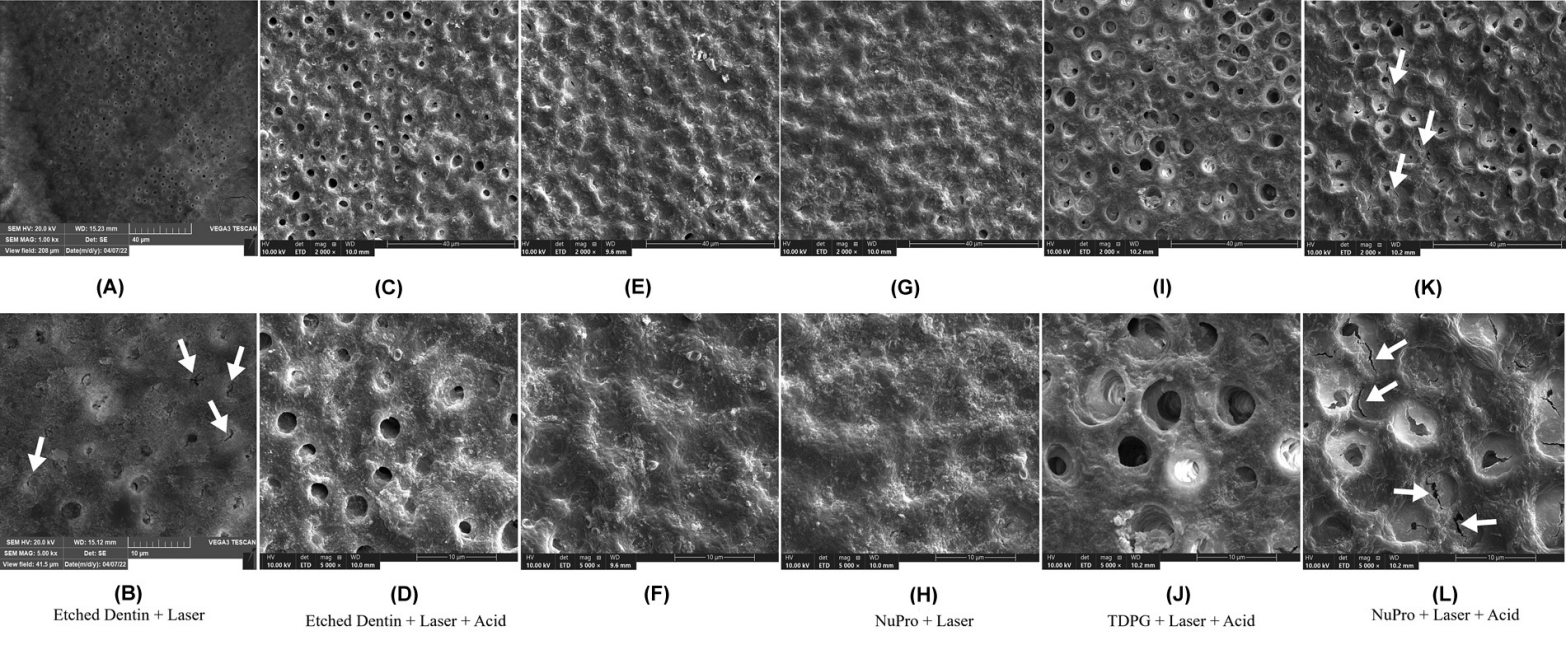
Representative SEM microimages under ×2,000– × 5,000 magnification showing samples of different groups treated with laser before and after acidic challenge with 6% citric acid: etched dentin ( **A–D** ), TDPG ( **E, F, I, J** ), NuPro ( **G, H, K, L** ). White arrows indicate laser-induced micro-cracks. Scale bar = 40 μm for ×2,000, 10 μm for ×5,000.

### Raman Spectroscopy

In sound dentin, the most prominent peak height at 960 cm ^−1^ band is assigned to the dentin mineral phosphate (PO4 ^3 −^ ), and the peak at 1,070 cm ^−1^ band is assigned to the mineral carbonate (CO3 ^2–^ ). The peak heights were processed in arbitrary units (a.u.). 27 Table 2 presents average Raman peak intensities for inorganic components (phosphate and carbonate) for different experimental groups before and after the acidic challenge. One-way ANOVA revealed statistically significant differences among the experimental groups for both phosphate and carbonate peak intensity ( *p* < 0.05) before and after acid challenge.

**Table 2:** Mean (SD) of Raman intensity (a.u.) for inorganic components for different experimental groups

	Before acidic challenge		After acidic challenge	
	Phosphate (960 cm ^−1^ )	Carbonate (1,070 cm ^−1^ )	Phosphate (960 cm ^−1^ )	Carbonate (1,070 cm ^−1^ )
Sound dentin	23,354 (4,256) ^A,a^	4,953 (2,729) ^D,j^	22,774 (4,218) ^a^	4,871 (2,745) ^j^
Etched dentin	11,724 (7,365) ^B,b^	1,676 (1,067) ^E,k^	16,170 (2,600) ^b^	2,600 (328) ^k^
Etched dentin laser	26,776 (10,759) ^A,c^	3,384 (788) ^D,l^	18,320 (3,006) ^c^	2,537 (848) ^l^
TDPG paste	10,141 (4,488) ^C,d^	1,507 (685) ^F,m^	12,820 (4,299) ^d^	1,864 (1,395) ^m^
TDPG paste laser	14,426 (4,282) ^C,e^	2,140 (617) ^F,n^	14,164 (2,693) ^e^	2,326 (403) ^n^
NuPro prophy paste	20,978 (3,880) ^A,f^	2,846 (692) ^G,o^	11,379 (2,491) ^g^	1,827 (316) ^o^
NuPro prophy paste laser	21,263 (12,016) ^A,h^	2,944 (894) ^G,p^	10,860 (2,659) ^i^	2,301 (808) ^p^

Abbreviation: a.u., arbitrary units.

Notes: Within a column, values with similar upper case superscript letters are statistically similar ( *p* > 0.05). Within a row, values with similar lower-case superscript letters are statistically similar ( *p* > 0.05).

#### Comparison of Raman Spectral Intensity before Acidic Challenge for Phosphate and Carbonate Peaks

Sound dentin showed the highest phosphate and carbonate peak intensities, serving as the mineral reference ( Table 2 , Fig. 4 ). Etching with 37% phosphoric acid produced a significant 49.8% reduction in both phosphate and carbonate relative to sound dentin. Laser treatment of etched dentin demonstrated the highest phosphate intensity of all groups, exceeding even sound dentin by 14%. TDPG paste (with or without laser) showed a moderate increase in phosphate and carbonate compared with etched dentin; further laser application improved the phosphate peak but not significantly.

**Fig. 4:**
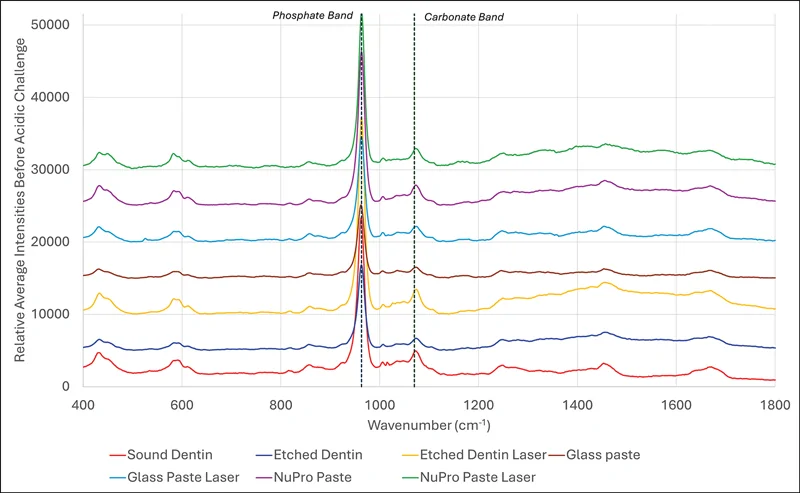
Relative average intensities before acidic challenge.

**Fig. 5:**
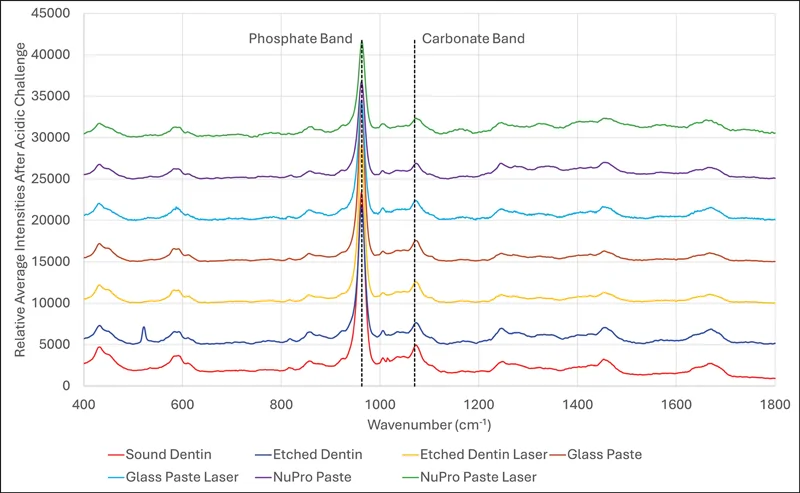
Relative average intensities after acidic challenge.

NuPro prophy paste, with or without laser, restored phosphate approaching 89.8% of sound dentin levels but did not outperform sound dentin or show additive laser benefit. For carbonate, NuPro prophy paste groups also improved over etched controls but remained below sound dentin.

#### Comparison of Raman Spectral Intensity after Acidic Challenge for Phosphate (960 cm ^−1^ ) and Carbonate (1,070 cm ^−1^ ) Peaks

Sound dentin maintained stable phosphate and carbonate levels, indicating strong acid resistance. Remarkably, etched dentin exhibited an unexpected 37.9% increase in phosphate intensity and a substantial 55.1% increase in carbonate after acid challenge. Laser-treated etched dentin showed a notable decrease in both phosphate and carbonate. TDPG presented a nonsignificant 21.6% increase in phosphate and 23.7% in carbonate intensities; while TDPG + laser combination was the most stable, with minimal loss after acid exposure. NuPro prophy paste groups experienced pronounced mineral loss, with significant drops in both phosphate and carbonate post-acid challenge, further reduced when combined with laser. A comparison of overall mineral content change post-acidic challenge across the different experimental groups is summarized in Fig. 6 .

**Fig. 6:**
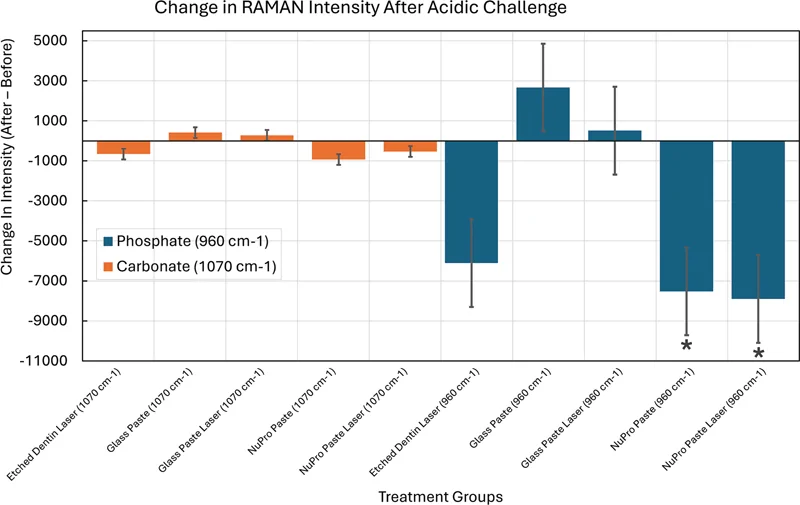
Change in RAMAN intensity for experimental groups post-acidic challenge.

## Discussion

The present study evaluated the effectiveness of TDPG paste and NuPro prophy paste, with or without diode laser irradiation, in occluding dentinal tubules and promoting dentin remineralization as a potential treatment for DH. Both null hypotheses were rejected: significant differences existed among treatment groups before and after acid challenge ( *p* < 0.05) for phosphate and carbonate peaks. According to the hydrodynamic theory, any biomaterial that can block the dentinal tubules and reduce the dentinal fluid conductance is thought to be useful in lowering the clinical symptoms of dentin hypersensitivity. 20 28 In terms of scanning electron microscopy, qualitative examination of representative specimens revealed that individual application of TDPG paste produced irregular crystalline deposits distributed nonuniformly across the dentin surface, with a few tubules remaining completely opened. In contrast, NuPro prophy paste showed more extensive surface coverage in the examined fields. These findings align with Milleman et al who reported that NuPro Sensodyne prophy paste achieved complete tubule occlusion after a single application, comparable to our findings. 29 Conversely, CPP-ACP products typically show more gradual, incomplete occlusion requiring multiple applications over weeks to achieve maximal effect. 30 31 A previous study evaluated the use of TDPG paste with Nd:YAG laser for dentinal tubules' occlusion, and reported that it was highly effective in sealing dentinal tubules, creating a smooth and homogenous surface layer, a key step for the management of DH. 19 Both TDPG paste + laser and NuPro prophy paste + laser combinations achieved complete tubule sealing with characteristic melted, wavy surface morphologies completely concealing underlying dentin structures. This observation confirms synergistic thermal-chemical interactions enhancing paste penetration and substrate bonding. 24 32 For this purpose, diode laser irradiation was used in this study as it could enhance the interaction between dentin and TDPG, facilitating deeper infiltration of glass particles into the dentinal surface, which could result in more effective and uniform remineralization. NuPro prophy paste's composition, which is primarily based on sodium fluoride (NaF), sodium silicate, monosodium phosphate, sodium carboxymethylcellulose, demonstrated remineralizing capabilities. According to Milleman et al, this formulation releases sodium ions, increases pH in the local environment, and facilitates the incorporation of additional phosphate and calcium ions into tooth structure, leading to favorable results in dentin remineralization and dentinal tubule occlusion. 29 The bioactivity of TDPG depends on its ability to release calcium and phosphate ions that participate in apatite precipitation. 11 However, this remineralization process is time-dependent and influenced by factors like pH and local ion availability. In short exposure times, the extent of ion-mediated occlusion may be limited, leading to incomplete tubule sealing. The components of NuPro prophy paste, including fluoride, can provide rapid occlusion through the immediate formation of mineral-like phases in the form of calcium fluoride and fluorapatite. 8 33

Following the acidic challenge, samples treated with both pastes showed extensive exposure of dentinal tubules with some remnants scattered on the surface, indicating that neither pastes provide sufficient resistance against acidic exposure when applied alone.

The resistance to acidic challenge was better in the samples treated with NuPro prophy paste and diode laser, as less exposure of dentinal tubules and a more intact and uniform surface layer was evident compared with the TDPG paste with diode laser treatment. However, micro-cracks were observed overlying the tubule's opening and the intertubular regions in these samples. This may be attributed to the stability of the NuPro prophy paste matrix during thermally induced crystallization following laser application and its ability to form fluorapatite crystals that reduce solubility in acidic environments. It should be noted that SEM observations were qualitative and based on a limited number of representative samples; therefore, the findings should be interpreted as morphological trends rather than statistically representative results. The present SEM findings ( Figs. 2 and 3 ) illustrate differences in the pattern and stability of tubule occlusion among the tested materials; however, SEM only reflects local surface morphology rather than functional sealing. Machado et al showed that morphological tubule occlusion does not always correspond to reductions in dentin permeability, since hydraulic conductance testing assesses overall fluid resistance under pulpal pressure. 34 In our study, although permeability was not measured, the morphological differences suggest variable durability and sealing potential. The results, therefore, support the view that SEM and quantitative permeability assessments are complementary techniques for evaluating dentin desensitizing efficacy. 34

The micro-Raman technique was used in this study to quantitatively assess the mineral content of dentin, based on the intensities of the phosphate (960 cm ^−1^ ) and carbonate (1,070 cm ^−1^ ) peaks for experimental groups after TDPG or NuPro prophy paste individual application and/or laser application before and after the acidic challenge. The intensity of these peaks correlates directly with the number of mineral molecules in the scanned volume, 27 allowing an accurate assessment of chemical composition before and after acidic exposure. The findings demonstrated distinct variations among the tested groups, highlighting the impact of etching, laser treatment, and the composition of different pastes on mineral retention.

As expected, sound dentin maintained a stable phosphate and carbonate intensities following the acidic challenge, reflecting its inherent mineral organization and resistance to acidic dissolution. Etched dentin, on the other hand, displayed a baseline decrease in both phosphate and carbonate intensity due to mineral loss from phosphoric acid treatment. However, following the acidic challenge, an unexpected increase in Raman peak intensities was observed. This may be explained by exposure of underlying mineralized zones, collapse of the organic matrix, or rearrangement of remaining apatite crystals, all of which could enhance the Raman signal.

The etched dentin with the laser group showed the highest phosphate intensity before acid exposure, surpassing even sound dentin. This high signal may be attributed to laser-induced surface crystallization of mineral phases, enhancing phosphate Raman scattering. However, this initial enhancement did not translate into long-term protection, as both phosphate and carbonate peaks declined sharply after acid exposure, indicating insufficient acid resistance.

When assessing the effects of TDPG paste, the Raman results revealed moderate mineral levels before acid exposure and a further increase after acidic challenge, especially in the TDPG paste group without laser. These findings were consistent with SEM images, which showed tubular occlusion and surface deposits, highlighting TDPG's remineralization potential even in acidic environments. 11 TDPG paste with laser treatment showed a smaller post-acid increase in carbonate peak, despite presenting a smooth, melted surface on SEM. This indicates that laser treatment may stabilize the applied material or improve paste-dentin bonding. 35 36 Moreover, it suggests that these materials may promote continued mineral deposition or undergo compositional changes in response to the acidic environment. It appears that laser application did not enhance mineral content beyond the TDPG alone, indicating a plateau effect or potential interference with ion diffusion during laser-induced heating.

The higher mineral content following acidic exposure in the TDPG groups could be due to several mechanisms that relate to the bioactive behavior of the paste. Under acidic conditions, the glass particles release calcium and phosphate ions, which can precipitate on the dentin surface as a calcium phosphate-rich layer. 37 This newly formed layer can subsequently crystallize into apatite-like structures, which results in elevated phosphate signal intensity in Raman spectra. Furthermore, the acid-induced surface roughness may expose more nucleation sites, promoting apatite crystal growth through available ions 38 and further contributing to the increase in mineral signal. In the NuPro prophy paste-treated group, an initially high phosphate likely results from the paste's fluoride and silicate composition, which promotes rapid formation of fluorapatite and phosphate-rich mineral phases on the dentin surface. Additionally, abrasive silica components remove the smear layer and expose fresh hydroxyapatite, further enhancing the phosphate signal. Post-acid challenge, NuPro prophy paste and NuPro prophy paste with laser groups showed significant reductions in phosphate and carbonate intensities. This suggests that although the paste formed a dense occlusive surface as seen under SEM, it lacked chemical resilience to acid dissolution. 39 Notably, NuPro prophy paste with laser showed the largest phosphate loss despite forming a uniform surface layer, which demonstrates that morphological sealing does not guarantee chemical protection. These findings support the conclusion that TDPG paste promotes a more chemically stable remineralization process, while NuPro prophy paste, though visually effective in SEM, may not offer sustained mineral integrity under erosive conditions. This indicates the importance of combining morphological and chemical analyses in evaluating occlusion materials for dentin hypersensitivity management.

This in vitro study has several limitations that should be considered before extrapolating to clinical practice. First, it lacks pulpal pressure and dentinal fluid dynamics present in vivo, which continuously challenge treatments and influence deposit degradation. Second, the study uses a single application and short-term evaluation, whereas clinical protocols typically involve repeated treatments over weeks to months, allowing better remineralization. The aggressive acid challenge used exceeds typical dietary exposure, which may limit clinical relevance. In addition, the small sample size in the SEM analysis limits quantitative tubule occlusion assessment, suggesting a need for future image analysis enhancements. Finally, complementary analytical techniques beyond SEM and Raman spectroscopy—such as confocal microscopy, elemental mapping, and microhardness testing—would strengthen the findings and better characterize treatment effects. Future research should focus on long-term TDPG remineralization studies over several weeks to assess mineral maturation and acid resistance. In situ and in vivo trials are needed to evaluate TDPG under natural oral conditions. Optimizing TDPG formulations, including TiO _2_ concentration and polymer vehicles, could improve efficacy. Alternative laser parameters and combination therapies with fluoride may enhance outcomes. Finally, clinical trials measuring patient pain and permeability testing will help confirm clinical effectiveness beyond laboratory findings. This study's primary novelty lies in demonstrating that TDPG exhibits pH-responsive bioactive behavior, continuing to release remineralizing ions and precipitate minerals even during acidic challenges.

## Conclusion

Within the limitations of this in vitro study, TDPG paste emerges as a favorable dentin remineralizing agent capable of enhancing mineral content under acidic conditions, while NuPro prophy paste offers rapid and uniform tubule occlusion. The combination of these materials with diode laser irradiation enhances initial dentinal sealing; however, only TDPG combined with laser maintains chemical stability and minimizes structural microdamage, suggesting superior durability.

For practicing clinicians, this suggests a dual strategy balancing immediate symptomatic relief using NuPro prophy paste + laser and sustained mineral reinforcement with TDPG-based therapies.

Future in vivo and in situ longitudinal studies are warranted to validate material performance under physiological conditions and over extended time frames, as the current work is constrained by short-term acid challenge and static in vitro modeling.

The study outcomes align with the tested null hypotheses by demonstrating significant differences in tubule occlusion and remineralization efficacy among treatment groups before and after acidic challenge. Nevertheless, cautious interpretation is advised, avoiding direct overgeneralization to clinical practice until further clinical evidence is available.

The integration of TDPG paste into existing desensitizing protocols offers a novel option for enhancing long-term tooth integrity with potential favorable cost-effectiveness, especially when coupled with portable, cost-efficient diode laser systems commonly found in dental offices. Durability challenges remain, particularly concerning TDPG paste alone under acidic conditions, underscoring the need for formulation optimization and extended clinical validation. In summary, these results provide a foundation for developing multifaceted, evidence-based approaches combining bioactive glass pastes and laser therapy to improve management of dentin hypersensitivity.
